# What can primary care services do to help First Nations people with unhealthy alcohol use? A systematic review: Australia, New Zealand, USA and Canada

**DOI:** 10.1186/s13722-020-00204-8

**Published:** 2020-08-18

**Authors:** Gemma C. Purcell-Khodr, K. S. Kylie Lee, James H. Conigrave, Emma Webster, Katherine M. Conigrave

**Affiliations:** 1 Faculty of Medicine and Health, Sydney School of Medicine; NHMRC Centre of Research Excellence in Indigenous Health and Alcohol, University of Sydney, Camperdown, NSW Australia; 2 Centre for Alcohol Policy Research, La Trobe University, Bundoora, VIC Australia; 3 Faculty of Medicine and Health, School of Rural Health, The University of Sydney, Dubbo, NSW Australia; 4 Drug Health Services, Royal Prince Alfred Hospital, Camperdown, NSW Australia

**Keywords:** Indigenous, First Nations, Alcohol, Primary health care, Outpatient, Relapse prevention medicines, Naltrexone, Disulfiram, Culture, Cultural healing

## Abstract

**Background:**

First Nations peoples of Australia, New Zealand, the United States of America (USA) and Canada are more likely to be non-drinkers than other people in these countries. However, those who do drink may be at greater risk of alcohol-related harms (at a population level) due to the ongoing impacts from colonisation and associated oppression. Addressing unhealthy drinking (drinking above recommended limits including alcohol use disorders) in primary care settings is one important way to increase accessibility of treatment.

**Methods:**

This systematic review identifies peer-reviewed studies of alcohol treatments delivered in primary care or other non-residential settings for First Nations peoples of Australia, New Zealand, USA and Canada. Literature searches were conducted in seven academic databases from their inception until March, 2020. We assessed evidence of treatment or implementation effectiveness, perceived acceptability or accessibility, and the study quality as assessed by the AXIS tool and by a measure of community participation in the research process.

**Results:**

Twenty-eight studies were included, published between 1968 and 2018. Studies reported on a range of alcohol treatments, from brief intervention to ambulatory withdrawal management, relapse prevention medicines, and cultural therapies. Brief intervention was the most studied approach. Cultural healing practices and bicultural approaches were a key theme amongst several studies. Four studies measured treatment effectiveness, including one randomised controlled trial (naltrexone vs naltrexone plus sertraline vs placebo) and two uncontrolled trials of disulfiram. Of the six implementation studies, three were (hybrid) effectiveness-implementation designs. Most of the remaining studies (n = 21) focused on treatment accessibility or acceptability. Community participation in the research process was poorly reported in most studies.

**Conclusions:**

Research evidence on how best to care for First Nations peoples with unhealthy alcohol use is limited. Trials of naltrexone and disulfiram presented promising results. Cultural and bicultural care were perceived as highly important to clinical staff and clients in several studies. More effectiveness studies on the full scope of alcohol treatments are needed. Greater community participation in research and more transparent reporting of this in study methods will be key to producing quality research that combines scientific rigour with cultural appropriateness.

## Background

Globally, unhealthy alcohol use is a leading cause of mortality and disability [1]. Unhealthy use includes the full spectrum of drinking above recommended limits, from hazardous alcohol use to severe alcohol use disorders. Harms from alcohol are exacerbated by colonial legacies which see First Nations peoples bearing a disproportionate burden of disease [1]. In this review, the term ‘First Nations’ will be used to respectfully refer to the distinct and diverse Indigenous tribal groups of all included countries, including Inuit and Métis peoples of Canada.

Alcohol consumption patterns in First Nations people of Australia, New Zealand, the United States of America (USA) and Canada have some similarities. Amongst these populations, data suggests a polarising trend to either abstinence or episodic heavy drinking [2–4]. For example, in Australia, Aboriginal and Torres Strait Islander peoples are more likely to abstain from drinking than their non-Indigenous counterparts [2]. However, as a population they are estimated to be 20–30% more likely to drink above recommended levels, and experience at least twice as many alcohol-related hospitalisations [5, 6]. These patterns do not represent individual vulnerability. There is large variability within and between communities [7, 8]. Complex sociocultural factors resulting from colonisation have exposed First Nations peoples to additional risks of harms from alcohol [9]. Factors include cultural hegemony (where the colonising culture dominates), racism, transgenerational trauma, unemployment, poor housing and disconnection from ancestral homelands, family and culture [9–11].

Primary care services can play an important role in helping people to change their alcohol consumption [12, 13]. In this systematic review, primary care is defined as the first point of contact with the health care system where generalist, holistic care is delivered, minimising the need for specialist care where safe to do so [14, 15]. There are a wide range of treatments available for unhealthy alcohol use in primary health settings [16, 17]. Brief interventions (BI) can be provided to individuals with hazardous or harmful drinking (non-dependent, or mild alcohol use disorders [AUDs]) [16]. For those with alcohol dependence (moderate-severe AUDs), motivational interviewing and other psychosocial therapies are available, though research on their effectiveness in primary care settings is limited [18, 19]. Where alcohol withdrawal management is necessary, home detoxification (ambulatory management) can be provided for carefully selected individuals [20, 21]. Pharmacotherapies such as naltrexone and acamprosate have been found to prevent relapse in combination with ongoing psychosocial supports and counselling amongst the general population in primary care settings [22]. These treatments offer promise for First Nations peoples, although minimal research has been done in this area [23, 24]. Those with severe alcohol dependence, or significant physical or mental health disorders or major social challenges, often require referral to a specialist service, and sometimes inpatient or residential care [16, 25].

For First Nations Peoples, the ability to receive treatment in primary health settings may be important due to several factors. For example, access to treatment ‘on Country’ (traditional homelands) or within one’s community is linked with spiritual, emotional and physical well-being [26–28]. Furthermore, kinship obligations (cultural caring) to children, partners and large extended families are a deterrent for individuals to travel for treatment, which for alcohol dependence may be for extended periods due to the relapsing nature of the condition. There are also financial barriers, including cost of transport, which may make residential facilities hard to access [29–31]. First Nations peoples may also fear discrimination in mainstream residential services [29, 32]. In addition, there is less stigma attached to attending primary care than to a specialised drug and alcohol unit [29]. From a health policy perspective, delivering alcohol care locally where it is appropriate, is also an economical and pragmatic solution to the chronic shortage of places in hospitals and residential rehabilitation services [33, 34].

Research on alcohol care in primary services for First Nations peoples has followed a similar trajectory as research on interventions for the general population. Historically abstinence-oriented approaches for treatment of alcohol dependence dominated [35]. From the 1980s a broader treatment philosophy arose, including earlier intervention and harm-reduction for those who cannot or do not want to change their drinking [35–37]. A greater understanding of the social determinants of health and the importance of ‘cultural competency’ or ‘cultural safety’ for First Nations peoples also has been embraced [38–40]. These two terms refer to ethical and effective conduct in cross-cultural settings, where the clinician’s own cultural values and worldview are acknowledged and implications of these (for interactions) are considered [41].

A recurring challenge in health research for First Nations peoples has been the epistemology underpinning research methods. In many cases the scientific approach has been at odds with Indigenous ‘ways of knowing’ [42, 43]. There is growing recognition of the need for culturally-informed studies, and for research methodology that has a decolonising frame [44, 45]. Involving First Nations communities in the co-design and co-production of research that impacts them is encouraged to meet ethical obligations and produce quality research [46].

There is a need to consolidate knowledge on the range of alcohol treatments available for First Nations clients in a primary care setting. In this paper we present a systematic review of the current evidence on treatments for unhealthy alcohol use in such settings. The aims were to: (1) quantify the number of peer-reviewed publications; (2) document the treatment approaches which have been studied (western or cultural) and assess evidence of their effectiveness; and (3) comment on the quality of the studies; both in terms of appraisal of study quality using a published scientific appraisal tool, and from assessing extent of community participation in the research process.

## Methods

A systematic search of peer-reviewed literature was conducted to determine what treatments are readily accessible in primary care to help First Nations individuals with unhealthy drinking.

The following electronic databases were searched on March 11, 2020: CINAHL, Psychinfo, Scopus, Informit, Medline, Web of Science and PubMed. Three other databases were also searched: Google Scholar, Australian Indigenous HealthInfoNet (including the Alcohol and Other Drugs Knowledge Centre) and the Lowitja Institute. Search terms are listed in Table 1, and included terms for population, country, substance, treatment and setting. Syntax and subject headings were adapted for each database as necessary. In one instance a set of search filters was applied to optimise search results in the PubMed database. These search filters are available at the Lowitja Institute [47] and were developed to link all variants of the term ‘Aboriginal’ with major topics in Indigenous (Australian) health. Additional records were identified by handsearching reference lists and consulting researchers from the field.

**Table 1:** Search strategy

Population			Country	Substance	Treatment		Setting	
Indigenous Aboriginal “First Nation*” “First people*” “Torres Strait Island*” Maori* Native* “Native American” “American Indian*” Indian* Inuit* Metis Alaska*	Aleut* Inupiat* Yuit Athabascan* Tlingit* Haida* Navajo* Cherokee* Arikara* Iroquois* Pawnee* Sioux* Apache* Comanche* Cree Ojibwa* Mohawk* Duwamish	Cheyanne* Blackfoot Seminole* Hopi Shoshone* Mohican* Shawnee* Mi’kmaq* Crow* Paiute Wampanoag* Ho-chunk* Chumash* Haida* Suquamish “Oceanic ancestry group”	Austral* Canad* North Americ* USA “United States of America” Americ* Alaska* New Zealand* Hawaii*	Alcohol*	Naltrexone Acamprosate Disulfiram Counsel* Men* group* Women* group* Culture Cultural approach* Cultural healing Traditional culture* Home detox* Healing circle*	Intervention* Program* Sweat lodge* Brief intervention* CBT DBT Cognitive behavioural therap* Cognitive behavioral therap* Dialectic behavioural therap* Dialectic behavioral therap* Relapse prevention medicine* Motivational Interview	Outpatient Primary care Primary health care General practice* GP* Doctor* Physician* Family practic* Medical practic* Medical center* Medical centre*	Aboriginal medical service Aboriginal health service “Aboriginal Community Controlled Health Service”

In applying the search strategy, “AND” was used between columns. “OR” was used within columns

### Inclusion criteria

Studies were included if: (1) they presented data on programs or interventions for reducing alcohol consumption or the harms caused by unhealthy alcohol use in First Nations individuals from Australia, New Zealand, United States of America, or Canada; (2) the program or intervention was aimed at individuals with known unhealthy drinking (i.e. above recommended limits ‘hazardous drinking’ or an alcohol use disorder), OR aimed at improving accessibility or acceptability of alcohol care in primary health care services; (3) the program or interventions were implemented in a primary care setting or other setting that was readily available to individuals in the community on an outpatient basis and; (4) data on First Nations participants was presented separately to other ethnic groups. Studies focusing on ‘Alcoholics Anonymous’ or similar mutual support groups were excluded as they are the subject of a separate review [48]. Studies which focused solely on screening were excluded. No language restrictions were implemented. Due to the dearth of research on this topic, no start date for the search was specified. Databases were searched from inception to 11 March 2020.

### Record screening

**Fig. 1:**
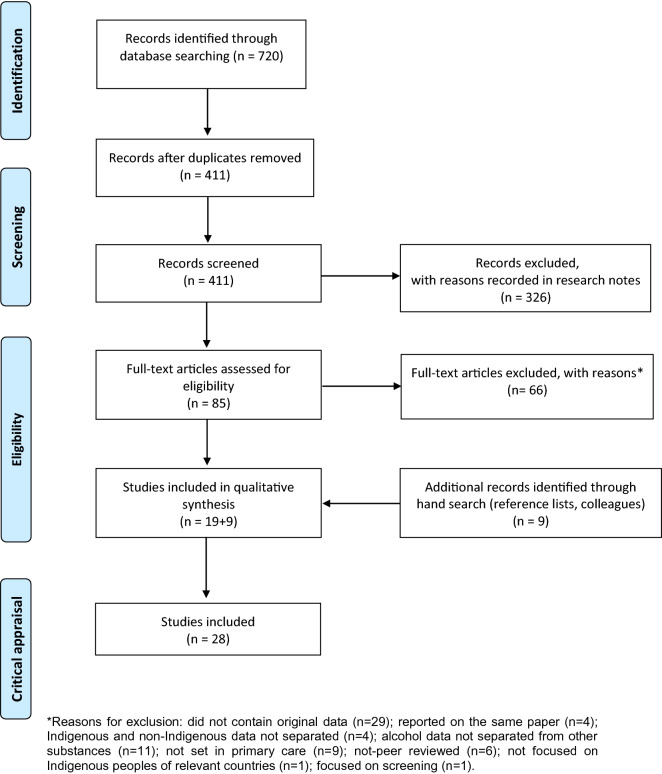
Preferred Reporting Items for Systematic Reviews and Meta-Analyses (PRISMA) flow diagram describing study selection

The search strategy was developed (GK, KC, KL) and then refined by two academic librarians. The search was conducted by one author (GK) and titles and abstracts of all retrieved articles were screened and reviewed independently by two authors (GK and KL). Discrepancies were discussed and resolved by consensus. Papers meeting inclusion criteria were subject to a full-text screen conducted independently by three authors (GK, KL, JC). Disagreements were debated and where unable to be resolved, an additional author adjudicated (KC). Reference lists of included articles from the full-text screen and articles recommended by colleagues were reviewed (GK) against the inclusion criteria. These additional papers were independently full-text screened (by GK and either KL or JC). No disagreements were found. A summary of the articles retrieved is presented in Fig. 1.

### Data extraction

Data extraction was completed by GK. Two authors (KL and JC) independently extracted data from half of the articles each. Care was taken to not allocate these authors any article on which they were a co-author. Data extracted included country and Indigenous population, participant characteristics, data collection methods, nature of the conditions treated, system used to categorise participants’ alcohol use, treatment strategy type (western [i.e. mainstream or non-Indigenous] or cultural), study focus (e.g. treatment effectiveness, implementation), length of follow-up, intervention details and outcomes. Studies that focused on implementation research were categorised according to a framework developed for the WHO [49], as either implementation or effectiveness-implementation design. The latter examines both the treatment effectiveness and implementation processes. ‘Western’ studies were defined as those that did not explicitly mention Indigenous cultural values or practices. ‘Cultural’ studies were grounded in traditional practices and philosophies of the respective First Nations participants. The three authors resolved discrepancies to form a combined version of data. Data extraction was independently checked (KC).

### Quality assessment

Study quality and risk of bias assessment was conducted using two tools: a version of the AXIS critical appraisal tool [50], which was later adapted by its authors to include assessment of the quality of randomised controlled trial (RCT) and cohort designs; and an adapted version of a tool to assess community participation in research [51–53]. The latter categorises First Nations community participation across seven levels, from no participation to self-mobilisation. Studies are classified on this scale based on participation at four time-points during research projects: diagnosis (identifying a community’s priorities), development (appropriate strategies to address the priorities), implementation (of the strategies) and evaluation (of the effectiveness of the project; Table 2). Scoring was conducted independently by three authors (GK, KL, JC). Discrepancies were discussed and resolved by consensus. Where participation data was not presented or unclear, an ‘unknown’ classification was allocated.

**Table 2:** Definitions of seven levels of community participation in the four phases of research project development [51–53]. Sourced from Snijder et al. [51], originally adapted from Pretty [52] and Wagemakers et al. [53]

Seven levels of community participation	Four stages of project development			
	Diagnosis	Development	Implementation	Evaluation
1. No participation	Completely top-down, community is not informed about or asked about issues in their community	Top-down, community is not informed about the development of the project	Top-down, community is not informed about the implementation of the project, only about activities they’re involved in	Top-down, community receives no information about evaluation
2. Passive participation	Outsiders decide on the issues that need to be addressed, community is informed	Outsiders control development, community is informed, but has no input	Outsiders control the implementation, community is informed, but has no input	Outsiders control the evaluation, community is informed, but has no input
3. Participation by information	Outsiders have control, community participates by providing information about their community. No feedback to the community and no checking for agreements	Outsiders have control over development, community potentially provides information about what they want, but outsiders don’t necessarily respond to this	Outsiders control implementation, community might provide information useful for implementation, but outsiders don’t necessarily listen to this	Outsiders control evaluation, community provides information through surveys and/or interviews, focus groups. Findings are not shared or checked for accuracy
4. Participation by consultation	Outsiders define problems and consult with community about their agreement, using outsider defined processes	Outsiders consult with community about potential projects to develop, but outsiders make final decision	Community participates in activities decided upon by the outsiders	Outsiders define evaluation process, community provides information and might make suggestions for improvement and feedback provided
5. Functional participation	Outsiders have predetermined goals and community assists in defining issues within those goals, outsiders make final decisions	Community works together with outsiders to develop projects decided upon by the outsiders.	Community and outsiders work towards implementation of projects, based on outsiders’ goals and processes	Community and outsiders work together in evaluation, based on goals as set by the outsiders
6. Interactive participation	Outsiders and community work together to identify the issues in the community and set goals for the project	Outsiders and community work together to develop suitable projects to address the agreed upon goals.	Community and outsiders implement the developed projects together, community has control and uses local resource	Evaluation methods are decided upon together and conducted in partnership
7. Self-mobilisation	Completely bottom-up, community identifies their own issues and sets their own goals, might contact outsiders to assist them where needed	Bottom-up, community makes decisions about project development, apply for funding and potentially contact outsiders where needed	Community implements projects, contacts outsiders for resources where needed, but remains in control over resources	Community conducts evaluations, potentially contacts outsiders for assistance, but stays in control over evaluation

## Results

Twenty-eight records met the selection criteria (Fig. 1). The included articles were published between 1968 and 2018, with the majority after 2010 (n = 19) [29–31, 54–69]. Most studies were conducted in Australia (n = 17) [29, 30, 54–62, 65, 67, 68, 70–72], followed by the USA (n = 7) [24, 31, 66, 69, 73–75], Canada (n = 2) [63, 64] and New Zealand (n = 2) [76, 77] (Table 3).

**Table 3:** Scope of the literature meeting inclusion criteria (n = 28)

Author (year)	Country (Indigenous population)	Primary focus of study^a^	Description of alcohol problem treated	Strategy: Western/cultural/both	Intervention/therapy studied
Treatment effectiveness					
Savard [75]^b^ (1968)	USA (Navaho)	Treatment effectiveness	Alcoholism	Western	Pharmacotherapy (disulfiram)^c^
Ferguson [73] (1970)	USA (Navaho)	Treatment effectiveness	Alcoholics	Western	Pharmacotherapy (disulfiram)^c^
O’Malley et al. [24] (2008)	USA (American Indian/Alaska Native)	Treatment effectiveness	Alcohol dependence	Western	Pharmacotherapy (naltrexone)
Venner et al. [69] (2016)	USA (American Indian/Alaska Native)	Treatment effectiveness	Substance use disorder and alcohol abuse/dependence	Both	MICRA (CBT)/cultural practices
Implementation research					
Kahn and Fua [72] (1992)	Australia (Aboriginal)	Effectiveness-implementation	Alcoholism	Western	Counsellor training as therapy
Clifford and Shakeshaft [59] (2011)	Australia (Aboriginal and or Torres Strait Islander)	Implementation research; staff and client acceptability	At-risk drinkers	Western	BI
Clifford et al. [61] (2013)	Australia (Aboriginal and or Torres Strait Islander)	Implementation research	At-risk of alcohol-related	Western	BI
D’Abbs et al. [62] (2013)	Australia (Aboriginal)	Effectiveness-implementation^d^	Alcohol problems	Both^e^	CBT/social-cultural support/pharmacotherapy (naltrexone)
Lovett et al. [67] (2014)	Australia (Aboriginal and Torres Strait Islander)	Implementation research	Problematic alcohol use	Both	Culturally appropriate introduction to BI and case management
Brett et al. [29] (2017)	Australia (Aboriginal)	Effectiveness-implementation^d^; client access; staff and client acceptability	Alcohol dependence	Western	‘Home detox’ (ambulatory withdrawal)
Treatment access and/or accessibility					
Hall [74] (1986)	USA (American Indian)	Client access; staff acceptability	Alcoholism	Cultural	Cultural practices
Brady et al. [70] (1998)	Australia (Aboriginal)	Staff acceptability	Alcohol problems	Western	BI
Huriwai et al. [76] (2000)	New Zealand (Māori)	Client acceptability	Alcohol problems	Cultural	Cultural practices
Robertson et al. [77] (2001)	New Zealand (Māori)	Staff acceptability	Alcohol problems	Cultural	Cultural practices
Brady et al. [71] (2002)	Australia (Aboriginal)	Staff acceptability and staff perception of client acceptability	Hazardous alcohol use	Western	BI
DeVerteuil and Wilson [63] (2010)	Canada (Aboriginal)	Client access; staff acceptability	Alcohol use problems	Both	Cultural practices
Panaretto et al. [68] (2010)	Australia (Aboriginal and Torres Strait Islander)	Staff perceptions of client access; staff acceptability	Alcohol abuse and alcohol harms	Western	BI
Allan [54] (2010)	Australia (Aboriginal and or Torres Strait Islander)	Staff access and acceptability	Problematic alcohol use	Western	BI
Gone [64] (2011)	Canada (Algonquian)	Client access; staff and client acceptability	Alcoholism	Both	Counselling/cultural practices
Allan and Campbell [55] (2011)	Australia (Aboriginal)	Client access and acceptability	Harmful substance use	Western	MI/BI/Counselling
Clifford et al. [60] (2012)	Australia (Aboriginal and or Torres Strait Islander)	Staff acceptability	Risky drinking	Western	BI
Conigrave et al. [30] (2012)	Australia (Aboriginal)	Client accessibility/awareness	Alcohol problems and alcohol use disorder	Western	BI
Legha and Novins [66] (2012)	USA (American Indian/Alaska Native)	Client access; staff acceptability	Alcohol abuse	Both	Cultural practices
Calabria et al. [57] (2013)	Australia (Aboriginal)	Client acceptability	Alcohol-related harms	Western	CBT (CRA + CRAFT)
Lee et al. [65] (2013)	Australia (Aboriginal)	Client access and acceptability	Alcohol use disorder	Both	Women’s group (cultural)
Brett et al. [56] (2014)	Australia (Aboriginal and Torres Strait Islander)	Staff perspective of treatment acceptability and accessibility	Alcohol dependence	Western	‘Home detox’ (ambulatory withdrawal)
Calabria et al. [58] (2014)	Australia (Aboriginal)	Staff acceptability	Alcohol-related harms	Western	CBT (CRA + CRAFT)
Hirchak et al. [31] (2018)	USA (American Indian/Alaska Native)	Client acceptability	Alcohol use disorders	Both	Contingency management/ cultural practices

^a^Studies are ordered in tables according to their focus and year of publication

^b^Results for this trial of disulfiram therapy were gathered from the background section of the cited publication (published 1964). The cited source for the data was an unpublished conference presentation by the same author who was involved with conducting the trial. Sufficient detail was presented to allow the methods to be described. This was cross-checked against a thesis by the same author. Data was not published elsewhere in refereed journals. Given the scarcity of quantitative data it was decided to include this study

^c^Both disulfiram trials required in-patient detoxification and commencement of therapy before participants were discharged to continue disulfiram therapy as outpatients. As part of the initial hospitalisation, after commencement of disulfiram, a “challenge dose” of alcohol was administered to measure the severity of reactions in a controlled environment. This is not standard practice today

^d^Primary focus was implementation but there were outcome results from a series of patients in these studies

^e^This intervention is mostly western, cultural care was planned but not delivered due to practical constraints

### Study characteristics

Studies examined treatments for a range of severity of unhealthy alcohol use, from hazardous use to alcohol dependence. Nine studies focussed on alcohol dependence or ‘alcoholism’ (*sic*) [24, 29, 56, 64, 69, 72–75]. Two studies focused on alcohol use disorders more broadly (severity not specified) [30, 31]. No study was found to exclude clients with dependence. Of the remaining 17 studies, a variety of terms were used to describe the nature of the alcohol problem being treated (Table 3). No diagnostic criteria or screening thresholds were provided for the majority (n = 19, 68%) of studies (Table 4). Of the 28 studies, almost half (n = 13; 46.5%) were quantitative [24, 55, 57, 61, 62, 69, 70, 72–77], nine were qualitative (32%) [31, 54, 56, 58, 60, 63, 64, 66, 71], and six were mixed methods (21.5%) [29, 30, 59, 65, 67, 68].

**Table 4:** Study methods and outcomes

Author (year)^a^	Participant characteristics	Classification of alcohol consumption	Study type	Length of follow-up	Client outcomes^b^: effectiveness or perceptions	Staff/service outcomes^b^
*Treatment effectiveness*						
Savard [75] (1968)	1) n = 30 alcoholic males 2a) n = 62 alcoholic males 2b) n = 39 non-abstinent, non-alcoholic males	ndp^c^	1) Follow up study of 30 disulfiram-treated alcoholics (*sic*) 2ab) Quantitative (cross-sectional)	1) 18 months 2ab) baseline interview only	1) decreased binge drinking and increased sober periods; 2ab) disulfiram is accepted excuse to decline alcohol and social pressures reduced (consumption not measured).	
Ferguson [73] (1970)	65 clan groups; 1) Intervention group n = 115 2) Comparison group n = 60	WHO ‘alcoholism’	Non-randomised controlled trial	6 months	Reduced incarceration; n = 50/115 sober 12–24 month following disulfiram therapy; sobriety not measured in controls.	
O’Malley et al. [24] (2008)	12 tribal groups; n = 68 American Indian/Alaska Native (AI/AN) participants	DSM-IV; CIWA-Ar	RCT	68 weeks	Significant decrease in alcohol-related consequences for naltrexone monotherapy vs placebo (p < 0.026)	
Venner et al. [69] (2016)	n = 8 members of one tribe	DSM-IV	Uncontrolled, pre-post study	8 months	Increase in days abstinent; decrease Addiction Severity Scores	
*Implementation research*						
Kahn and Fua [72] (1992)	n = 240 participants	ndp	Uncontrolled pre-post study	N/A^d^	n = 138/145 maintained sobriety post-graduation	
Clifford and Shakeshaft [59] (2011)	n = 32 health staff; n = 24 clients	ndp	Mixed methods, pre-post study	N/A		Increased staff confidence to deliver BI; increase documentation and delivery; high-risk drinkers resistant to alcohol referral
Clifford et al. [61] (2013)	n = 4 Indigenous health services n = total of 50 clients	2001 NHMRC guidelines	Uncontrolled, pre-post study	N/A		Increased BIs
D’Abbs et al. [62] (2013)	n = 19 clients; n = 30 quasi control; n = 32 program staff/other stakeholders	ndp	Trial with quasi-controls	N/A	n = 15/19 reported decrease or stop drinking post program contact. n = 21/30 quasi-control with similar result.	Implementation challenges incl: time constraints, staff turnover, GP hesitancy to prescribe naltrexone; strengths incl multidisciplinary care, flexibility
Lovett et al. [67] (2014)	n = 34 health service staff	ndp	Mixed methods: quantitative (cross-sectional); literature review	N/A		Proposed ‘yarning style’ BI; implementation challenges noted; staff least confident in BI when client not seeking help
Brett et al. [29] (2017)	Qual: n = 7 staff (1 GP, 1 GP trainee, 2 nurses, 3 Aboriginal DandA workers), n = 4 clients; n = 8 community stakeholders (incl. 4 Elders) Quant: n = 8 clients	2009 NHMRC guidelines	Mixed methods (cross-sectional)	N/A	Qual: clients rate program as accessible, streamlined and holistic; challenges also noted. Quant: n = 5/8 abstinent at 6-week follow up; n = 8/8 still engaged with supports. No major adverse events reported during detox	Qual: desired model principles incl. cultural safety, privacy (preventing community shame), keeping family together, peer support, accessible and streamlined. Feedback given on strengths and challenges of model as implemented
*Treatment access and/or acceptability*						
Hall [74] (1986)	n = 44 services^e^	ndp	Quantitative (descriptive)	N/A		n = 22 services incl. sweat lodge or encouraged use at external sites; n = 8 provided access to community-based sweat lodge; medicine man used on and off-site
Brady et al. [70] (1998)	n = 29 services	ndp	Quantitative (cross-sectional)	N/A		Aboriginal health services more likely to offer exclusive abstinence-based/Minnesota model of care; BI offered in half of services
Huriwai et al. [76] (2000)	n = 6 services^f^; total n = 105 clients	ndp	Quantitative (cross-sectional)	N/A	Clients rated strongly the importance of cultural elements in treatment	
Robertson et al. [77] (2001)	n = 90 alcohol and drug-user treatment services; n = 217 staff	ndp	Quantitative (cross-sectional)	N/A		Strong support for cultural interventions with Māori clients
Brady et al. [71] (2002)	n = 8 health care workers; n = 6 general practitioners; n = 25 clients	AUDIT (tns^g^) and 2Q’s on consumption	Qualitative (not clearly specified)	18 months		5/6 doctors still using BI
DeVerteuil and Wilson [63] (2010)	n = 7 services^e^; total of n = 24 frontline staff; n = 1 staff member identified as Aboriginal	ndp	Qualitative (service case study)	N/A		n = 6 services refer for off-site cultural activities; n = 1 service has on-site cultural programs (incl. sweat lodge accessible by non-residents)
Panaretto et al. [68] (2010)	n = 4 health services; total of n = 46 staff	ndp	Mixed methods (cross-sectional)	N/A		n = 3/4 services offered BI in past 12mths; challenges noted
Allan [54] (2010)	n = 47 staff (DandA workers; primary health care workers)	ndp	Qualitative (action research)	N/A		Conflicting approaches to care between staff
Gone [64] (2011)	n = 4 current/former administrators; n = 4 counsellors; n = 11 clients^h^	ndp	Qualitative (ethnography)	N/A		Program philosophy was based on medicine wheel and spiritual elements of AA; positive client experiences documented
Allan and Campbell [55] (2011)	n = 149 Aboriginal people attending community events; n = 16 sewing group participants; n = 5 DandA and Aboriginal health workers	ndp	Uncontrolled pre-post study	N/A	Strong client engagement and client acceptability	
Clifford et al. [60] (2012)	n = 5 ACCHSs; total of n = 37 health staff	ndp	Qualitative (descriptive)	N/A		Scepticism of BI effectiveness and outcomes
Conigrave et al. [30] (2012)	n = 47 participants	AUDIT score of 8 +	Mixed methods (cross-sectional)	N/A	Participants unaware of outpatient treatments e.g. ambulatory withdrawal and medicines	
Legha and Novins [66] (2012)	n = 18 substance abuse treatment programs serving AI/AN communities (representing 3 tribes across 7 states); n = 77 service providers (n = 22 clinical admin staff; n = 55 frontline staff)	ndp	Qualitative (grounded theory)	N/A		Cultural beliefs/values core to program; adapted western models used
Calabria et al. [57] (2013)	Clients of an ACCHS or DandA service n = 110 Indigenous; n = 6 non-Indigenous but have Indig. spouse or child	AUDIT (tns)	Quantitative (cross-sectional)	N/A	Strong client acceptability ratings	
Lee et al. [65] (2013)	n = 21 staff; n = 24 female Aboriginal clients	AUDIT-C score of 4 +	Mixed methods cross-sectional survey; qualitative (descriptive)	N/A	Participant self-esteem and identity improved	
Brett et al. [56] (2014)	n = 4 Indigenous health services; n = 1–3 staff at each service	2009 NHMRC guidelines	Qualitative (descriptive)	N/A		Feedback for/on implementation of outpatient detox
Calabria et al. [58] (2014)	n = 19 DandA treatment agency staff; n = 3 ACCHS health staff	ndp	Qualitative (not clearly specified)	N/A		Tailoring process is documented and feedback gathered for adapting the counselling and counsellor certification process and improving feasibility
Hirchak et al. [31] (2018)	n = 61 participants (incl. individuals with AUDs, treatment providers, and community members)	ndp	Qualitative (not clearly specified)	N/A	Rated culturally acceptable	

^a^Studies are ordered in tables according to their focus and year of publication

^b^These columns contain the outcome data, qualitative or quantitative with regard to the type of participants (clients or staff and services) included in the study

^c^No definition provided

^d^Not applicable

^e^All services were residential. Study was included as sweat lodge available for outpatients on-site or in a community-based facility

^f^Study included data from residential and outpatient services. Only outpatient service data was included

^g^Threshold score not specified

^h^Service offered residential and outpatient programs with facilities also open to broader community. All data included is relevant to outpatient settings

Study interventions, primary outcomes, and analytic strategies were highly varied. We classified studies according to their research focus (Table 3). Two-thirds (n = 18) of the studies focused on treatment accessibility or acceptability from the perspective of clients (n = 6), staff (n = 8) or both (n = 4) [30, 31, 55, 57, 65, 76]; [54, 56, 58, 60, 68, 70, 71, 77]; [63, 64, 66, 74]. The remaining one-third focused on treatment effectiveness (n = 4) [24, 69, 73, 75]; effectiveness-implementation (n = 3) [29, 62, 72] or implementation research (n = 3) [59, 61, 67]. One effectiveness-implementation study also evaluated client access and staff access and acceptability [29], and another study evaluated implementation and client and staff acceptability [59].

The majority of studies (n = 17; 60%) reported on western treatment approaches [24, 29, 30, 54–61, 68, 70–73, 75], with only 3 reporting specifically on cultural approaches (n = 3) [74, 76, 77] for addressing unhealthy alcohol use. Eight studies reported on both [31, 62–67, 69]. The balance of western compared to cultural approaches varied by country. Of the Australian publications, the majority (n = 13/17) examined use or implementation of a western approach, most often BI [30, 54–61, 68, 70–72]. The two studies from New Zealand solely focused on the importance and acceptability of culture in alcohol care [76, 77]. Half of US-based studies (n = 4/8) included cultural approaches [31, 66, 69, 74]. The two Canadian studies explored bicultural approaches [63, 64].

### Study outcomes

#### Treatment effectiveness outcomes

Seven studies in total measured alcohol consumption (e.g. frequency of ‘binges’ and duration of abstinence) [24, 29, 62, 69, 72, 73, 75]. These were the only studies that examined effectiveness, and include both treatment effectiveness and effectiveness-implementation studies (Table 3).

Of these seven studies, one was a double-blind, randomised-controlled trial (RCT) of naltrexone alone and in combination with sertraline, conducted with Alaska Natives [24]. Alcohol-related consequences were significantly lower (38%) in the naltrexone monotherapy group compared with placebo (72%; *p *= 0.026). There was a high abstinence rate in both the naltrexone only and the placebo group over 68 weeks (percentage days abstinent of 96.6% ± 2.9% vs 88.8% ± 3.0% respectively). Differences between the two naltrexone conditions were not significant on any measure. However, the small sample (n = 68), may not have given sufficient power to detect moderate effect sizes.

Two of the older studies were trials of disulfiram on Navaho country with several clan groups. In both trials, patients were administered an alcohol challenge in hospital after starting disulfiram. The first, in 1968, reported over half of the participants (n = 16/30) maintained abstinence at 6–18 months after detoxification and commencement of disulfiram. Another seven participants (n = 7/30) had relapsed but resumed disulfiram therapy and were again abstinent at the post-intervention assessment [75]. Study participants reported disulfiram as helpful in avoiding social pressure to drink. The second trial of disulfiram in 1970, reported that nearly half of participants (n = 50/115) were abstinent at 12–24 months [73]. During the 18-month treatment period the number of arrests for drunkenness (*sic*) decreased by 78% compared with the prior 18 months. Neither study had a true control group and alcohol consumption was not measured in their comparison groups.

Two studies trialled interventions in Australian Aboriginal Community Controlled Health Services (ACCHS). The first (uncontrolled) study examined a multi-disciplinary alcohol intervention, including naltrexone [62]. However, the untreated comparison group reduced their drinking at similar rates to the participants who received the intervention. The second study documented the development of a ‘home detox’ (ambulatory withdrawal management) program and the outcomes with n = 8 clients [29]. The program typically ran for 5 days with daily administration of diazepam (weaning dose) and thiamine in combination with counselling and a relapse prevention plan. Home visits and transport were provided. At the 6-week follow up over half (n = 5/8) of the clients remained abstinent (including one who had transitioned to residential rehabilitation). All eight clients were still engaged with service supports. No major adverse events were reported during detox.

The (non-random, uncontrolled) ‘counsellor training as therapy’ study [72] reported a high rate of abstinence at 24 months (95% of participants). Individuals with known past ‘very severe alcohol abuse’ were nominated to take part in the training by community leaders or existing treatment program staff. Potential participants were deemed eligible based also on their current interest, level of motivation and perceived capacity to complete the program. Lastly, an uncontrolled pilot study that used a culturally adapted form of MICRA (Motivational Interviewing and Community Reinforcement Approach) [69] reported an increase in days abstinent and a decrease in addiction severity scores (using a culturally adapted version of the Addiction Severity Index, ASI-NA) at 8-month follow-up compared to the pre-treatment period and baseline respectively.

#### Client awareness or perceptions

Client awareness or perceptions were investigated regarding three domains: acceptability of a treatment approach; awareness of treatments and perceived accessibility; and opinions on the importance of cultural elements in care, and documentation of cultural treatment content.

Three Australian studies assessed and documented the acceptability of an intervention with clients [29, 55, 57]. Tailoring to optimise cultural appropriateness and acceptability was conducted in two other studies [31, 58]. Both of these interventions were reported as acceptable after tailoring. Community-based education and group brief intervention was piloted in one study with Aboriginal Australians [30]. In this study, participants were found to be unaware of non-residential treatment options such as ambulatory withdrawal management and relapse prevention medicines. Client acceptability and accessibility of one such non-residential option were explored in a separate Australian study from the perspective of staff and service providers [56]. Staff perceived ambulatory withdrawal management as a viable and underutilised approach, and feedback for optimal implementation was documented (Table 4). In a third Australian study focusing on ambulatory withdrawal [29] (described in the treatment effectiveness section), participants reported being ‘satisfied’ or ‘very satisfied’ with the program, would repeat it if needed, and would recommend it to a friend. Clients in this study reported preferring ACCHS over mainstream services. Cultural appropriateness was a core aim in developing the pilot program of this western treatment.

Two studies explored cultural practices in treatment, one with First Nation Americans and the other with Māori peoples [64, 76]. Clients and clinical staff rated cultural elements as highly important in both studies (Table 4).

#### Staff and service outcomes or perceptions

Fourteen studies included data on treatment implementation outcomes (implementation studies) or staff perceptions of the acceptability and accessibility of alcohol treatments [29, 54, 59–61, 63, 64, 66–68, 70, 71, 74, 77]. Most of these studies examined brief intervention (BI) (n = 6/14) [59–61, 67, 68, 71]. Training and outreach support were found to increase staff confidence to deliver BI [59] and resulted in increased BI delivery rates [61]. Two studies reported on perceived barriers to delivering BIs. These included: scepticism of BI effectiveness [60]; time pressure in the clinical environment, and high staff turnover [68]. It was also noted in a survey of (n = 29) Australian services, that service philosophy (abstinence as a goal versus moderated drinking) dictated BI delivery [70]. Lastly, one study described implementation of a culturally-tailored BI [67].

Five studies presented data from staff and clients on service delivery of cultural or bicultural treatment approaches [63, 64, 66, 74, 77]. Of these, three studies documented the importance of cultural interventions; this included two staff surveys [66, 77] and one case study [64]. The surveys reported: strong support from non-Indigenous and First Nation clinicians to use cultural interventions to increase client wellbeing and engagement [77]. Also, that services with cultural beliefs at their core (i.e. holistic views of health; spiritual care; importance of kinship etc.) could implement traditional healing interventions alongside western approaches when those western approaches had been tailored for the local First Nations communities [66]. The case-study described a largely cultural model of care centred on the medicine-wheel philosophy [64]. However, this treatment approach also included spiritual elements of Alcoholics Anonymous. The two other studies investigating service delivery of cultural and bicultural interventions reported the ‘sweat-lodge’ as the most frequently used therapy [63, 74]. Other cultural activities were also described at one residential service which allowed access for out-patients [63]. Staff attitudes towards the integration of cultural healing interventions within a standard western-treatment facility varied. Some staff at that service expressed scepticism and devalued cultural elements in care. This was most frequently expressed by staff in facilities which did not provide sweat-lodge access on-site but outsourced cultural activities to community organisations [63].

### Study quality

There were only two controlled trials (one blinded RCT [24] and one non-randomised controlled trial [73]). The remainder of the quantitative studies had no control group (n = 5) [55, 61, 62, 69, 72]. Most studies were of moderate quality when examined with the AXIS critical appraisal tool [50].

Objectives and aims were clearly described, and study design was appropriate for the stated aims in two-thirds of studies (n = 25, 89%; n = 20, 71% respectively) [24, 29–31, 55–61, 63–74, 76, 77]; [24, 29–31, 54, 56–60, 63–68, 70–77]. Study populations were clearly defined (n = 25, 89%) and taken from samples which were likely representative in most studies (n = 23, 82%) [24, 29–31, 54, 57–70, 72–77]; [24, 29–31, 54, 57–63, 65–68, 70, 72–77]. However, most studies (n = 25, 89%) did not describe non-responders or missing data [24, 29–31, 54–59, 61–65, 68–77]. Methods were insufficiently described in a third of studies (n = 10, 36%) to allow replication [30, 31, 54–56, 62, 64, 66, 71, 75]. Basic data (e.g. demographics) were not described in nearly half of studies (n = 13, 46%) [54–56, 59–64, 70, 71, 74, 75], of which, nine were published in the last decade [54–56, 59–64]. Discussion and conclusions did not appear justified by the results in a quarter of studies (n = 6, 21.5%) [24, 54–56, 61, 69] and ethical approval was not mentioned in just under a third of studies (n = 9, 32%) [54, 63, 64, 70–75].

### Extent of community participation

Community participation by First Nations peoples varied (Table 5). The highest levels of participation (Levels 5 to 7) were found in both the ‘diagnosis’ phase of the research, and the ‘development’ phase, with nine studies (32%) each [24, 29–31, 62, 65, 69, 73, 75]; [29–31, 62, 64, 65, 69, 73]. This was followed by the ‘implementation’ phase (n = 7; 25%) [29–31, 62, 64, 65, 73]. Four studies scored highly in all of the first three phases (14%) [30, 31, 62, 73]. Only one study scored highly in all four phases [29]. Participation was described with insufficient detail to be assessed in twelve studies (43%) [56–61, 63, 67, 68, 74, 76, 77].

**Table 5:** Level of community participation across the development of each study

Author (year)	Four stages of project development			
	Diagnosis	Development	Implementation	Evaluation
Treatment effectiveness				
Savard [75] (1968)	5–6	–^a^	–	–
Ferguson [73] (1970)	6	4–5	4–5	–
O’Malley et al. [24] (2008)	5	4	4	–
Venner et al. [69] (2016)	6	5	4	4
Implementation research				
Kahn and Fua [72] (1992)	3	–	3	–
Clifford and Shakeshaft [59] (2011)	–	–	–	–
Clifford et al. [61] (2013)	–	–	–	–
D’Abbs et al. [62] (2013)	7	7	7	4
Lovett et al. [67] (2014)	–	–	–	–
Brett et al. [29] (2017)	6–7	6	7	6
Treatment access and/or acceptability				
Hall [74] (1986)	–	–	–	–
Brady et al. [70] (1998)	1	1	1	1
Huriwai et al. [76] (2000)	–	–	–	–
Robertson et al. [77] (2001)	–	–	–	–
Brady et al. [71] (2002)	3	3	3	–
DeVerteuil and Wilson [63] (2010)	–	–	–	–
Panaretto et al. [68] (2010)	–	–	–	–
Allan [54] (2010)	3	–	–	–
Gone [64] (2011)	–	4–5	5	–
Allan and Campbell [55] (2011)	2	–	–	–
Clifford et al. [60] (2012)	–	–	–	–
Conigrave et al. [30] (2012)	6	6	6	–
Legha and Novins [66] (2012)	2	2	–	–
Calabria et al. [57] (2013)	–	–	–	–
Lee et al. [65] (2013)	7	6	5	4
Brett et al. [56] (2014)	–	–	–	–
Calabria et al. [58] (2014)	–	–	–	–
Hirchak et al. [31] (2018)	5	5	5	4

^a^Insufficient data to apply assessment criteria

## Discussion

This is the first systematic review of treatments for unhealthy alcohol use for First Nations peoples that are feasible in or from primary care settings. Publications from Australia, New Zealand, Canada, and the USA were considered. The 28 included studies report on all aspects of standard alcohol treatment, from brief intervention to ambulatory withdrawal management, relapse-prevention medicines, as well as on cultural treatments. Between countries, we found Australian studies were heavily focused on early intervention for non-dependent drinkers while in the USA there was greater emphasis on interventions for alcohol use disorders, including dependence. Terminology used to classify participant alcohol use varied. Only seven studies attempted to measure intervention effectiveness. Of these, there was only one RCT. Accordingly, insight into what approaches lead to the best clinical outcomes for First Nations clients is limited. Furthermore, quality was negatively impacted by low levels of community participation in research processes and often a lack of transparency in this regard. Large effectiveness studies, conducted in close partnership with communities, are required to inform best practice with these populations.

### Western approaches

Western approaches were studied in a variety of ways. Some trialled the standard western treatment approach, while others engaged First Nations clients or staff in tailoring it for the Indigenous context. Western approaches were also used in conjunction with cultural therapies, sometimes as a bicultural strategy, and other times the cultural elements were outsourced to community organisations as ‘clip-on’ elements of care.

Brief intervention was the most studied approach; however, no study measured its effectiveness. Effective client–clinician communication is at the core of BI and is likely impacted by the communication preferences and cultural protocols of First Nations peoples [78, 79]. The one study which implemented a culturally-influenced version of BI [67], did not describe the development of the approach. Tailoring and implementation studies are needed to optimise BI delivery and should record client perceptions of acceptability, as well as effectiveness.

Ambulatory withdrawal management was examined in two Australian studies in relation to Aboriginal Community Controlled Health Services. For carefully selected individuals, this approach may help increase accessibility and acceptability of withdrawal management. The research also demonstrated how community partnerships can be a part of developing, running and evaluating such programs within a primary health care service.

A small number of studies trialled alcohol relapse-prevention medicines. The RCT of naltrexone reported significant reduction in alcohol-related consequences compared to placebo despite low participant numbers [24]. Inclusion criteria for the trial may have been a barrier for First Nation people’s participation as interested persons were required to provide confirmation of “tribal enrolment” or a “Certificate of Indian Blood”. Of the 28 included studies, this was the only study where verification of First Nations status was discussed. This may have been due to the study’s exploratory aim of looking at predicted responses to treatment based on genetic traits. Consultation with community partners may help to respectfully decide on terms of participation and reduce barriers to participation in clinical trials.

The two other studies involving relapse prevention medicines trialled disulfiram. Both were performed half a century ago (1968 and 1970) and involved administration of a test dose of alcohol to precipitate a reaction. This is not standard practice today and would raise ethical concerns. Due to the scarcity of relapse prevention medicine trials with First Nations peoples, it was decided to include these studies. Disulfiram is not as frequently prescribed as it once was, due to concerns over serious adverse reactions in the event of relapse to drinking and debate over its effectiveness, particularly with unsupervised dosing. Despite these concerns, disulfiram is still prescribed internationally for carefully selected and voluntary patients [80]. In Australia, lack of government subsidies on the cost has also been identified as a barrier to its use [81]. The models of support offered alongside disulfiram therapy in these two studies appeared to be culturally tailored and progressive for their era. However, given the studies were conducted over 50 years ago, client acceptability data may need to be re-examined. Acamprosate, was not trialled or mentioned in any study. In contrast, amongst the general population both acamprosate and naltrexone have been trialled extensively.

Underutilisation of these medicines is a population-wide trend, although a range of social determinants may see First Nations peoples being less likely to access alcohol pharmacotherapy than non-Indigenous populations [82, 83]. Based on the included studies, medicines to prevent relapse seem acceptable within a culture-informed framework [24, 56]. Methods to increase awareness of the medicines and their availability through primary care services, could be considered in future research [30].

### Cultural and bicultural approaches

Cultural healing practices and spiritual customs were a major theme in this review. Traditional healing approaches have also appeared in other areas of First Nation’s health research, responding to the communities’ call—“our culture is our treatment” [17, 42, 84, 85]. The integration of cultural protocols and traditional therapies into primary health care has been associated with the movement for cultural reclamation. These themes were highlighted in one of the earliest included studies [74]. Along with innovations in treatment content and tailoring of delivery to increase acceptability and accessibility for minorities, community leaders are pushing for control over what is studied and how research questions are framed [86]. First Nations Elders are resisting the framing of research in deficit-based rhetoric, in favour of strength-based approaches and stories of resilience [87–89]. With this paradigm shift comes opportunities to create knowledge that incorporates the best of western, evidence-based practice with traditional knowledges, forming a third stream valuable in both spheres. This concept has been articulated in “two-eyed seeing” and “Ganma” philosophies [90, 91], from Canada and Yolngu Nation (Australia) respectively.

The notion of Indigenous culture in health care is tied to holistic definitions of health. This invariably involves connectedness to family, traditional homelands, spirituality, community identity and reciprocal relationships [26, 92, 93]. In research of the general population, factors such as connectedness have also been identified as important on the recovery journey for addictions [94, 95]. Likewise, connectedness has been identified as a protective factor against AUDs by First Nations peoples [66, 86, 96].

Overarching health policy has recognised the need for culturally-informed and tailored health care [38–40, 97]. However, cultural and bicultural therapies are still largely unconsidered in the national alcohol treatment guidelines of all four included countries [16, 25, 98–100].

### Study quality

The quality of the included studies was assessed from two lenses—from a (western) scientific lens and from a community values lens. We found reporting was more complete for scientific measures. Privacy concerns for smaller study sites may account for the lack of basic demographic data. Over half of the studies collected data through qualitative interviews and observations. While qualitative methods are important in understanding participants’ thoughts and feelings, it does not allow quantification of effectiveness. Limitations also apply to pre-post treatment comparison studies, as individuals are most likely to enrol in a trial when they are highly motivated or at a point of crisis. This can falsely inflate intervention success rates, as the participants may have already been on a path to abstinence or moderated drinking (regression to the mean). While there was only one RCT, strict conditions maintained in the study environment may not hold up in many routine clinical settings. In addition to further efficacy trials, studies establishing the effectiveness of interventions for improving unhealthy alcohol use outcomes in real world settings are also needed.

Almost half of the studies did not report on community participation. This is concerning as the value of community engagement (even outside community-based, participatory study designs) is well recognised and promoted through research ethical guidelines [46, 101, 102]. According to these guidelines, best practice with First Nations communities involves meaningful engagements, where all parties are consulted before the ‘problem’ is defined and well before study aims and designs are planned. Reporting details of the tailoring process may be challenging due to journal word constraints. Until this is a standard requirement of reporting, onus is placed on researchers to succinctly describe the processes undertaken.

It is clear from the included studies, there is often a divergence of beliefs (ontology), value systems (axiology) and ways of knowing what is truth (epistemology), between the scientific and First Nation communities. This is particularly apparent with the qualitative data identified in this review. An individual’s or community’s experiences, perceptions and opinions (anecdotal evidence) rate low in the scientific pyramid of evidence. Meanwhile, this type of evidence has an important place in *Indigenous ways of knowing* [42]. Future research collaborations with First Nations communities will have to navigate these differences to generate knowledge that is valuable in both knowledge bases (pluralism).

### Implications

The small number of controlled trials and effectiveness studies identified in this review, is one example of the pressing need to build on community partnerships. For example, First Nations communities have expressed concern over ethical issues with RCT designs (withholding beneficial treatment from one part of the community) [103]. Alternative study designs such as the ‘stepped-wedge trial’ and ‘wait-list control’ have been approved as culturally safe by several communities [86, 104]. Given the cultural diversity within First Nations tribal groups, tailoring of treatment approaches to suit local cultural beliefs, values, protocols, and communication preferences is encouraged to promote client engagement with primary care services. Future studies could also consider attitudes to alcohol as a factor influencing treatment acceptability or delivery. Such attitudes may vary greatly between and within First Nations communities. Some communities may have a strong preference for abstinence as goal, based on past community experience of alcohol’s harms, desire to avoid risk behaviours arising from non-Indigenous culture, or missionary influence [6, 105]. For such communities, the idea of a controlled drinking goal (for non-dependent drinkers or for community) may be controversial [70]. Anecdotal evidence suggests in many communities (Indigenous and non-Indigenous) that there is a high awareness of the social harms from alcohol, but less awareness of its health effects. Any treatment efforts may need to be accompanied by sensitively conducted, community-based health promotion around alcohol.

To ensure comparability between studies, improved reporting of inclusion and exclusion criteria is needed, for example the thresholds used to define unhealthy drinking or alcohol use disorders (i.e. DSM or ICD criteria, or screening questionnaire score). Lastly, many promising treatment approaches have been abandoned after a single descriptive study, so there are many potential treatment approaches to build on and study.

## Limitations

This review did not include grey literature due to practical constraints. There is likely documentation of cultural and western treatments with First Nations peoples in that literature which has been missed. Additionally, approaches such as ‘managed alcohol programs’ were not within the scope of this review, but have been reported to offer some promise in improving health and wellbeing outcomes [106, 107]. Community participation quality ratings may not be a true reflection on all studies’ actual conduct as there may be more engagement than was recorded.

## Conclusions

Overall this area is under researched with very few treatment effectiveness studies conducted, and on a limited range of therapies. Much work is needed to define approaches to unhealthy alcohol use that are most effective and acceptable for First Nations clients in a primary care setting. Trials of naltrexone and disulfiram yielded promising results, and acamprosate is yet to be studied. Cultural and bicultural approaches were generally delivered by Indigenous-specific health services or mainstream services with strong links to a First Nations community. These were found acceptable, but effectiveness outcomes have not yet been studied. Taking a strengths-based approach through community partnerships will be an essential next step if we are to produce quality research that combines scientific rigour with cultural appropriateness. First Nations researchers and Indigenous research methodologies will be an important part of this process.

## Data Availability

Data for this project is stored at the University of Sydney, Camperdown 2006 New South Wales, Australia.

## Abbreviations

AI/AN: American Indian/Alaska Native
DandA: Drug and alcohol
AUD: Alcohol use disorder
USA: United States of America
DSM-IV: Diagnostic and Statistical Manual of Mental Disorders, 4th edition
BI: Brief intervention
MICRA: Motivational interviewing and community reinforcement approach
ACCHS: Aboriginal Community Controlled Health Service
ASI-NA: Addiction Severity Index adapted for use with Native Americans
CIW-Ar: Clinical Institute Withdrawal Assessment-alcohol (revised version)
RCT: Randomised controlled trial
NHMRC: National Health and Medical Research Council (Australia)
WHO: World Health Organization
AUDIT: Alcohol Use Disorders Identification Test
ndp: No definition provided
N/A: Not applicable
tns: Threshold score not specified
GK: Gemma Khodr
KL: Kylie Lee
JC: James Conigrave
KC: Katherine Conigrave
